# Beyond facilitation and inhibition: a configurational mechanism study of cognitive transitions in human–AI collaboration

**DOI:** 10.3389/fpsyg.2026.1821188

**Published:** 2026-04-29

**Authors:** Xiaohui Shao, Weizheng Jiang, Khairul Nizam Osman

**Affiliations:** 1 Faculty of Business, Accounting, Finance, Law & Humanity (FOBAFLH), MAHSA University, Kuala Lumpur, Malaysia; 2 School of Management, Wuhan Technology and Business University, Wuhan, China; 3 School of Management, Wuhan University of Science and Technology, Wuhan, China

**Keywords:** cognitive transitions, fsQCA, human–AI collaboration, knowledge conversion, technology-supported education

## Abstract

Ongoing debates in higher education regarding whether artificial intelligence should be further integrated or deliberately constrained call for empirical research that offers a more explanatory analytical framework. However, existing studies on the human-AI collaboration (HAC) paradox are largely grounded in a binary logic of facilitation versus inhibition, leaving the dynamic mechanisms underlying complex cognitive processes insufficiently explored. To address this issue, this study adopts a dialectical perspective on explicit and tacit knowledge in knowledge conversion, and systematically examines the mechanisms of cognitive conflict, regulation, and equilibrium underlying cognitive transitions in HAC. Drawing on data collected from 316 participants in an authentic instructional context, this study constructs a configurational model incorporating social interaction (SI), tacit knowledge acquisition (TKA), internalization (I), self-motivation (SM), and trust in AI (TiAI), and employs fuzzy-set qualitative comparative analysis (fsQCA) to examine multiple equifinal pathways leading to higher-level cognition. The findings identify two distinct types of driving mechanisms underlying cognitive transitions: a high human-centered engagement pathway in the absence of AI, and a compensatory pathway in which AI offsets deficiencies in human-centered conditions. These results suggest that cognitive transitions emerge not from the linear effect of isolated factors but from the dynamic counterbalancing and configuration of psychological characteristics and technological conditions. In this specific educational context, AI functions as a mediating and compensatory agent that mitigates cognitive imbalance. Methodologically, this study demonstrates the logical compatibility between fsQCA and knowledge spiral; theoretically, it extends the explanatory boundaries of the HAC paradox; and practically, it provides evidence-based guidance for the structural deployment of AI support in higher education.

## 1 Introduction

In recent years, artificial intelligence (AI), driven by its powerful generative capabilities, has rapidly transformed learning and teaching models in higher education on a global scale. The 2025 AI in Education Report (Microsoft, 2025) reflects Microsoft’s overall optimism regarding the future prospects of AI applications in education. Specifically, the report indicates that AI has been widely adopted across different educational stages, accompanied by high levels of satisfaction among students, educators, and educational administrators. Against this backdrop, one of the central tasks facing contemporary educational systems is to align with this trend by systematically establishing AI literacy training programs. The underlying rationale is that, within educational contexts, AI is no longer perceived solely as a functional tool but increasingly assumes characteristics of a peer-like entity. Guided by educational objectives that precede market demands, students are expected to approach AI from a managerial perspective, learning to conceptualize and coordinate its functional allocation in learning and task execution. Moreover, AI is widely regarded as playing a critical role in stimulating creative thinking and enhancing collaborative processes. Such optimistic views have also been documented in earlier studies (e.g., Roll and Wylie, 2016; Chen et al., 2020).

Despite the urgency with which these perspectives articulate an optimistic vision of integrating technology into education, practical evidence suggests that such expectations may be overstated. Drawing on technology acceptance theory, existing studies have identified inhibitory effects within human-AI collaboration (HAC), indicating that these effects arise from misaligned cognitive trust, which in turn leads to excessive human reliance on AI (Jiang et al., 2025a). Deeper AI involvement may further introduce the risk of cognitive outsourcing. Based on survey interviews, Gerlich (2025) reports a significant negative association between frequent AI use and the development of critical thinking. Moreover, a substantial body of research converges on concerns that AI may undermine human creativity, albeit for different reasons. For example, Fan et al. (2025) argue that AI intensifies metacognitive laziness, whereas Ferrara (2024) contends that AI-generated misinformation contributes to the erosion of truth.

Based on the foregoing discussion, an urgent question emerges under this prevailing trend: what criteria should guide decisions regarding the comprehensive introduction or deliberate restriction of AI in higher education? The dual effects associated with AI in fact reflect a broader paradox confronting much of contemporary management research. In this context, the primary challenge for educational practice does not lie in hastily determining whether AI should be adopted.

Rather, the more fundamental research question (RQ) concerns: How human cognitive development can be aligned with AI intervention in a manner that maintains a regulable and recoverable dynamic balance.

Existing research exhibits clear limitations in explaining this paradox. Given the extensive and fragmented body of studies addressing paradoxes in HAC, this discussion is organized around several recurrent themes. First, research on the tension between trust in AI and reliance on AI (e.g., Bedué and Fritzsche, 2022; Riley and Dixon, 2024) has tended to overemphasize the risks arising from trust misalignment, while offering limited examination of the regulation of deeper cognitive processes. Second, studies on cognitive outsourcing and capability degradation (e.g., Buçinca et al., 2021; Gerlich, 2025) largely remain at the level of phenomenological description, without further unpacking the underlying configurations of psychological conditions or the dynamic processes that generate these outcomes. In addition, another prominent theme concerns AI and creativity (e.g., Zhang et al., 2021; Ferrara, 2024). Although this stream of research frequently addresses the double-edged effects of AI on creativity, it remains primarily confined to a tool-oriented perspective and fails to analyze the substitutive or complementary effects between human-centered factors and technological intervention.

To deepen the understanding of this paradox, this study adopts a dynamic equilibrium perspective to examine the interaction between human cognitive processes and AI intervention. Situating the analysis within the context of higher education, the study demonstrates that AI not only functions as a tool influencing human cognition, but also, to some extent, reshapes cognitive pathways and information-processing patterns. By characterizing and interpreting this complex process, the study aims to advance understanding of the diverse developmental trajectories emerging in AI–HI (Artificial Intelligence–Human Intelligence) integration.

To this end, this study employs the fuzzy-set Qualitative Comparative Analysis (fsQCA) method to identify multiple equifinal pathways arising from different configurations of conditions. By emphasizing multiple equifinal configurations of causal conditions, fsQCA offers distinct advantages in capturing the complexity and dynamism of cognitive development within HAC (Pappas and Woodside, 2021). This study seeks to elucidate the complex generative mechanisms underlying human-AI symbiosis in higher education and to provide a theoretical foundation for leveraging AI support while simultaneously avoiding cognitive degradation.

## 2 Theoretical framework and research questions

### 2.1 Higher-level cognition (HLC) in cognitive transitions

Bloom’s taxonomy of cognitive objectives represents one of the classical theoretical foundations for distinguishing between lower-level and higher-level cognition. According to Bloom’s taxonomy, memory and comprehension are classified as lower-order cognition, whereas analysis, evaluation, and creation are categorized as higher-level cognition; application is positioned as a transitional category between lower- and higher-level cognition. The acquisition of higher-level cognition (HLC) is one of the key manifestations of cognitive transitions, and this concept has also been applied to HAC (Herrmann-Werner et al., 2024). Bloom’s taxonomy provides an operational framework for categorizing cognitive levels in HAC, enabling both the characterization of AI’s enhancement effects on cognition and the fine-grained measurement of cognitive processes.

Building on prior research (Jiang et al., 2024, 2025a, 2025b), AI has been shown to exert differentiated effects across distinct cognitive levels, and these effects are significantly associated with individual human characteristics. Existing studies further demonstrate, from a quantitative perspective, that Bloom’s taxonomy more readily maps onto task contents at different cognitive levels and allows for finer-grained capture of human-AI collaborative task processes, such as the substitution of AI for repetitive tasks (e.g., memory-related tasks) (Jiang et al., 2025a).

The process through which individuals achieve cognitive transitions is equivalent to a cycle in which collective tacit knowledge is first explicated and subsequently re-tacitized at the individual level—namely, the knowledge spiral. According to SECI model^1^ (Nonaka and Yamaguchi, 2022), knowledge conversion is conceptualized as a dynamic process whereby collective cognition flows into individual cognition, followed by individuals’ reorganization, processing, and innovation of that cognition, which is then re-externalized, forming a continuously advancing closed loop. Against the backdrop of rapid AI development, the cognitive interaction process within AI-human intelligence (AI-HI) collaboration has become a more pervasive and salient manifestation of the knowledge spiral. This process is influenced not only by AI-related factors such as computational power and algorithms (Zhang et al., 2025b) but also by individual attributes, including skills and subjectivity (Jiang et al., 2025b). Moreover, the SECI model can serve as a theoretical foundation for assessing cognitive dynamics. For instance, Sian Lee and Kelkar (2013), drawing on the SECI framework, provide a clear comparison of the application of digital intelligent tools across different stages of cognitive acquisition.

From a process-oriented perspective on cognitive development, Bloom’s taxonomy should not be understood as a static hierarchy of abilities but rather as a framework that captures the ongoing processes through which individuals continuously reorganize and create knowledge within task contexts. This progressive logic is consistent with the mechanism of the knowledge spiral. Specifically, lower-order cognition primarily involves the reproduction and comprehension of existing explicit knowledge, whereas advancement toward HLC requires individuals to reconstruct and internalize knowledge by integrating cognitive strategies, agency, and related factors. This progression substantively corresponds to the continuous transformations described in the knowledge spiral.

Therefore, we argue that the process of achieving cognitive transitions encompasses analysis, evaluation, and creation, all of which belong to HLC. This process is not the result of a linear improvement in the capabilities of a single actor. In HAC contexts, non-linear factors, such as technological support, collective knowledge spillovers, and individual behaviors, jointly shape the attainment of HLC. Moreover, the SECI model and Bloom’s taxonomy exhibit an inherent logical consistency in their depictions of cognitive development. Their integration enables a vertical representation of the acquisition of HLC while simultaneously offering a horizontal view of knowledge flows between humans and AI. On this basis, drawing on the coupling of these two theoretical perspectives, this study proposes an operational framework for quantifying the process through which HLC is attained in HAC.

### 2.2 Key psychological processes in human–AI collaboration (HAC)

#### 2.2.1 Social interaction (SI) and cognitive triggers

From the perspective of human team collaboration, effective social interaction (SI) is not only a prerequisite for meaningful HAC but also a critical psychological trigger for cognitive transitions. Within the socialization and externalization stages of the SECI model, tacit experience, human intuition, ethical reasoning, and contextual understanding can be transformed into explicit forms through contemporary visualization and intelligent technologies (i.e., Molina et al., 2024). In this process, interaction behaviors and modes do not directly generate new knowledge; rather, they activate individuals’ capacities for application, analysis, and reflection, thereby creating the conditions necessary for subsequent cognitive reorganization and transitions.

Within a context that conceptualizes AI as a special form of intelligent agent, SI scenarios can be extended into a multi-actor interaction structure involving human–AI–human dynamics. According to the review by Xi et al. (2025), an AI agent is defined as an artificial entity capable of perceiving its environment, making decisions, and subsequently taking actions. Across a range of important investigative and analytical tasks, AI has increasingly been regarded as a “human partner” that can participate in discussions, offer suggestions, and generate feedback (e.g., Kim and Im, 2023; Deng et al., 2025). Compared with human-to-human communication, effective HAC is more likely to elicit reflection and revision. Owing to its advantages in terms of high interaction frequency, low cost, and immediacy, such collaboration can substantially amplify cognitive triggering effects (Goethe University Frankfurt am Main et al., 2023).

However, effective SI is not simply grounded in mechanisms of information triggering; rather, it is constructed through processes of comparison, questioning, and collaborative sense-making, which in turn give rise to psychological processes of cognitive dissonance and asymmetry. When individuals interact with others or with AI and are exposed to divergent viewpoints and modes of thinking, the calibration of others or AI facilitates the disruption of existing cognitive schemas. Critical thinking and disruptive metacognitive strategies are more likely to generate cognitive tension during interaction (Rivas et al., 2022), thereby prompting a reexamination of the meaning of learning.

Accordingly, SI in HAC should not be understood as mere information exchange but rather as a critical psychological process through which individuals, via cognitive dissonance and reflective calibration, trigger the explicitation of knowledge.

#### 2.2.2 Tacit knowledge acquisition (TKA) and cognitive input

In the process of cognitive transitions, high-quality cognitive input is a critical prerequisite for the attainment of HLC. Research in knowledge management suggests that high-quality cognition largely derives from valuable inputs of tacit knowledge (i.e., Kucharska and Erickson, 2023). Tacit knowledge is characterized by vague and elusive details that are difficult to articulate; in this context, it is often resistant to textualization and structural representation. Psychological phenomena such as experience and intuition thus become the primary carriers of tacit knowledge exchange, while playing a pivotal role in complex problem solving and highly creative execution activities. The transfer of tacit knowledge relies on bidirectional transmission and is constrained by factors such as personality, learning needs, and motivation (Juan, 2025).

Within HAC contexts, the transfer of tacit knowledge has expanded toward multi-source and multimodal forms. Compared with traditional modes of transmission, technology-driven approaches to TKA through virtual technologies do not represent a fundamental departure; rather, collaborative information technologies (CITs) constitute an extension of the bidirectional nature of knowledge transfer (Priego-Roche et al., 2016). By contrast, AI technologies, through learning from large-scale data, historical cases, and patterned structures, are increasingly intervening in human interaction processes in a humanoid-like form that approximates “subjective experience” (Okamura and Yamada, 2020). Moreover, the transformative impact of AI is further reflected in its capacity to extend the boundaries of perceiving, comparing, and absorbing tacit knowledge through advances in algorithms and computational power (He and Burger-Helmchen, 2025).

The transfer of tacit knowledge is not a passive form of input. Drawing on research on tacit knowledge sharing (Holste and Fields, 2010; Juan, 2025), a broad consensus has emerged that subjective conditions such as motivation, willingness, and trust effectively facilitate the conversion of tacit knowledge. This prerequisite is equally applicable to HAC contexts. Existing studies have shown that effective human-AI interaction benefits from individuals’ conditional trust in AI (Jiang et al., 2025a) as well as from a corresponding level of mastery or control over AI (Jiang et al., 2025b).

In conclusion, tacit knowledge functions as an “input source” in cognitive transitions. Its value does not depend on the degree to which information is made explicit, but rather on whether it can activate the cognitive resources required for subsequent analysis, evaluation, and creation.

#### 2.2.3 Internalization (I) and cognitive structure reorganization

Tacit knowledge can give rise to high-level cognitive transitions only when it is stably absorbed by individuals and embedded within their cognitive systems. According to Nonaka and Yamaguchi (2022), internalization constitutes a critical mechanism for the emergence of ideas and the enhancement of creativity. Its essence lies not in simple information aggregation or repetitive practice, but in cognitive restructuring and deeper psychological dynamics manifested through individuals’ subjective intentions and strategies.

From the perspective of cognitive restructuring, HAC significantly influences individual internalization. First, by providing immediate feedback and intelligent reasoning, AI enhances the dynamism and structural characteristics of knowledge during the tacitization process, thereby strengthening learners’ capacities for self-critique and self-correction (Bharatha et al., 2024; Ritala et al., 2024). More critically, AI reshapes existing human cognitive strategies and action pathways. By reducing repetitive cognitive processing and offering both procedural guidance and emotional support, AI enables greater cognitive investment in the construction of conceptual meaning, the development of framing strategies, and reflective sense-making. Related studies, such as Rasouli et al. (2022), suggest that social robots undertake complex tasks in intervening in social anxiety, thereby supporting and complementing the work of clinical practitioners.

From a Gestalt perspective, the internalization process in HAC can be understood as individuals’ pursuit of a “good form” under conditions of external disturbance. When AI is introduced as an intelligent agent into meaning-making cognitive practice tasks, it disrupts the original balance of cognitive organization and induces the reconfiguration of new cognitive schemas (Garfinkle, 2025). Inconsistencies in knowledge combinations drive Gestalt transformations, rendering internalization analogous to insight-based cognitive leaps. In this sense, internalization is no longer a unidirectional process of individual absorption and transformation; rather, it more closely resembles a structural nexus through which AI, as a form of “heterogeneous input,” is converted into human “system-level performance.”

In short, internalization function as a critical nexus linking cognitive structures and system-level performance within HAC, serving as a core psychological mechanism that drives cognitive restructuring and enables cognitive transitions.

#### 2.2.4 Self-motivation (SM) and psychological regulation

According to prior research (e.g., Afzal and Crawford, 2022), self-motivation (SM) often serves a regulatory role in sustaining behavioral engagement and psychological change. As a critical psychological variable underlying personal goal attainment and academic success, SM involves stimulation derived from the task itself and the intrinsic enjoyment that energizes action, thereby strengthening goal-directed persistence. In the process of cognitive transitions, higher levels of SM enable individuals to sustain greater cognitive load and emotional tension. Conversely, in the absence of such psychological regulation, individuals are more likely to avoid deep cognitive processing, resulting in cognition remaining at lower-order levels (Zhang et al., 2021).

Theoretical perspectives on SM can likewise be extended to HAC contexts, where its regulatory function shapes both the manner and depth of human–AI interaction. Studies by Li et al. (2025) and Yang et al., 2025, among others, have incorporated SM as a moderating factor. Collectively, these studies reveal that highly motivated individuals tend to regard AI as a positive medium for self-reflexive practice, achieving the realization of self-fulfilling prophecies through repeated dialogue, interrogation, comparison, verification, and continuous self-monitoring. Moreover, SM effectively regulates individuals’ cognitive persistence when confronted with cognitive conflicts arising from uncertain AI feedback or erroneous prompts. Drawing on higher education contexts, Hong and Huang (2025) argue that both internal and external factors within self-determination mechanisms significantly influence university students’ interactions with AI.

In summary, as a sustainable mechanism of psychological regulation, SM substantially shapes the depth and stability of individuals’ engagement in HAC.

#### 2.2.5 Trust in AI (TiAI) and calibration

We have conducted a systematic examination of trust relationships in HAC and argue that the formation of trust in AI (TiAI) is complex and non-linear (Jiang et al., 2025a). Specifically, excessive transparency within cognitive trust significantly undermines autonomous thinking and reflective capacity, whereas functionality trust (FT) and emotional trust (ET) exert positive effects on the acquisition of HLC. Notably, no significant interaction effects are observed between these two forms of trust. Accordingly, we emphasize the necessity of continuous regulation and calibration of AI. Under such psychological conditions, rational calibration of AI is manifested in individuals’ ability to dynamically adjust their engagement with AI in accordance with task complexity and their own capabilities. From a cognitive perspective, AI primarily serves as a form of support rather than a substitute for decision-making (Okamura and Yamada, 2020). This calibration mechanism enables individuals to maintain a sense of mastery over AI, preserving active control over problem structures and solution pathways, thereby preventing cognitive outsourcing from eroding the internalization process.

In sum, trust in or reliance on AI does not operate in isolation; rather, it facilitates the formation of HLC through calibration mechanisms. This constitutes an essential complementary condition for sustaining effective and symbiotic AI-HI integration.

### 2.3 Knowledge conversion and cognitive transitions in human–AI collaboration

#### 2.3.1 The logic of knowledge conversion

In contemporary higher education, assessment is no longer confined to outcomes alone (i.e., Microsoft, 2025). Particularly in the AI era, AI’s capacity to generate standardized answers far exceeds that of individuals. The core mission of university education is therefore to guide individuals to make independent judgments and assume responsibility for their consequences even in contexts characterized by the absence of standard answers, uncertain feedback, or deliberate constraints on AI use (e.g., Herrmann-Werner et al., 2024). In the pursuit of this goal, uncertainty, cognitive conflict, and psychological risk are likely to emerge. However, when this process is decomposed into the conversion between tacit and explicit knowledge, it becomes more conducive to capturing the uncertainty, conflict, and risks inherent in cognitive transitions.

Prior studies (Canonico et al., 2020; Zhang et al., 2025b) suggest that knowledge conversion involves both the externalization of tacit knowledge and the internalization of explicit knowledge. In team co-creation contexts, a complete cognitive process depends on the effective conversion between these two forms of knowledge (i.e., Canonico et al., 2020). Specifically, metaphors emerging in interaction contribute to the construction of structured explicit knowledge, while such systematized and consensual cognition requires the reshaping of individuals’ prior cognitive structures through practice and strategic adaptation.

**Figure 1:**
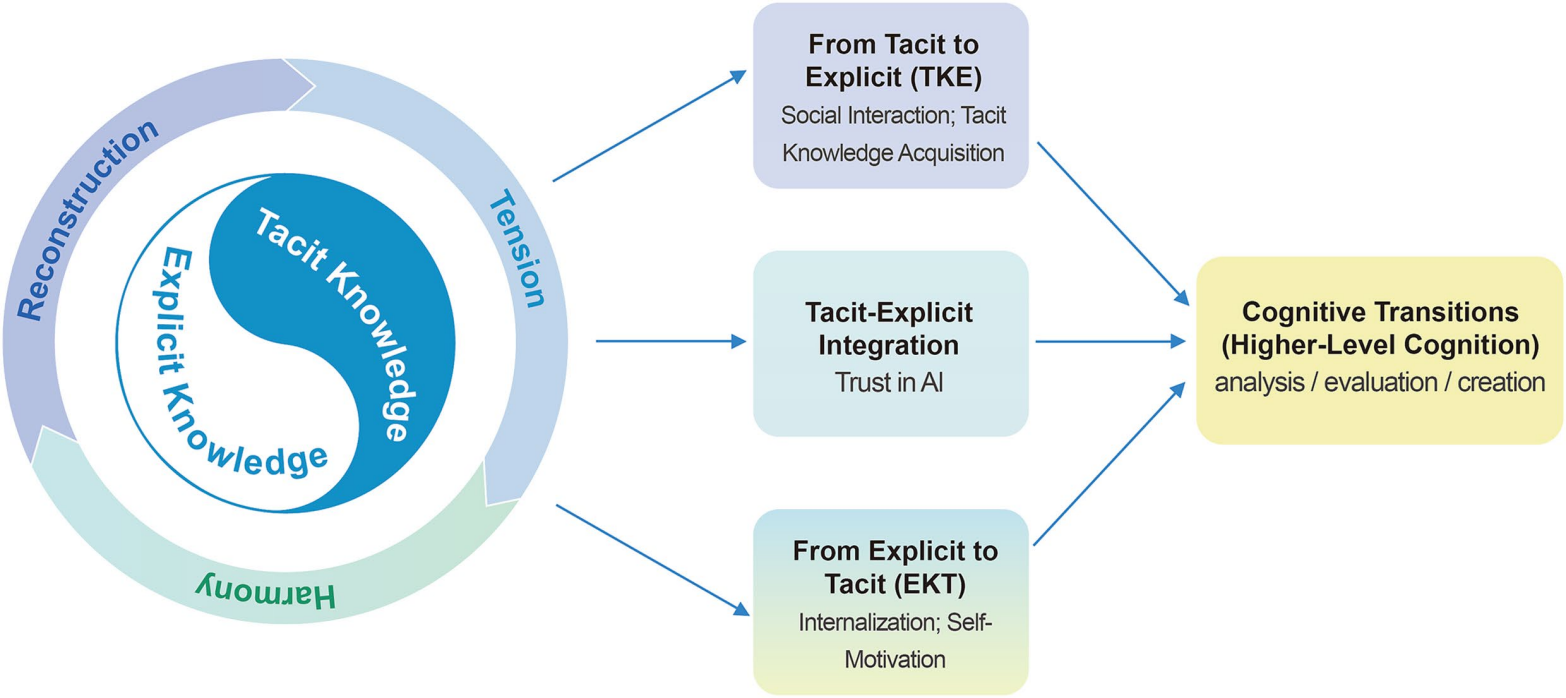
Tacit–explicit knowledge conversion framework of cognitive transitions in human-AI collaboration.

Accordingly, this study conceptualizes cognitive transitions in HAC as a continuous process of conversion between tacit and explicit knowledge across different stages. This process is inherently non-linear and involves the interaction of multiple psychological mechanisms (i.e., Glikson and Woolley, 2020), including cognitive conflict, reflective regulation, and structural reconfiguration. In this context, which reflects the tension, harmony, and reconstruction inherent in the spiral process of knowledge (Chin et al., 2025). This perspective provides a useful lens for uncovering the underlying mechanisms of cognitive development in HAC (see Figure 1).

#### 2.3.2 Tacit knowledge explicitation (TKE): social interaction (SI) and tacit knowledge acquisition (TKA)

Tacit knowledge explicitation (TKE) is closely associated with social interaction and metaphorical processes, through which tacit and unstructured knowledge is transformed into explicit and articulable forms via both formal and informal communication. In this process, consistent with the notion that “we know more than we can tell” (Polanyi, 1966), individuals need to expose themselves to diverse perspectives and problem-induced tensions in order to successfully articulate and represent their understanding.

SI, as a triggering condition and problem-initiating mechanism, provides a foundational basis for TKE. Through dialogue, questioning, and feedback with others and with AI, deeply embedded cognitive experiences of the self are continuously activated. At the same time, TKA functions as an input source in the form of incomplete propositions, entering the cognitive system through vague perceptions, contextual judgments, or unstable understanding. When tacit knowledge is repeatedly invoked and contrasted within interactive contexts, the latent components of self-cognition are gradually transformed into states that are open to commentary and reflection.

In short, TKE does not occur spontaneously; rather, it is jointly driven by SI and TKA. This process marks the point at which self-cognition begins to be externalized and enters a task stage that is open to reconstruction.

#### 2.3.3 Explicit knowledge tacitization (EKT): I (internalization) and SM (self-motivation)

Explicit knowledge tacitization (EKT) is largely shaped by individual-level factors. Through self-determination and the effective enactment of strategies, individuals reintegrate explicit knowledge into internal and stable structures, thereby achieving cognitive schema reorganization or cognitive leaping. The core task of this process is the absorption, selection, and consolidation of structured explicit knowledge, with the aim of forming cognitive resources that can be readily mobilized by the individual.

At this stage of knowledge conversion, internalization constitutes the central task. Through internally driven activities such as repetitive application, construct comparison, and reflection, individual internalization embeds concepts, patterns, and principles into the cognitive structure, allowing them to function without reliance on external prompts. In parallel, SM plays a regulatory role in the internalization process, manifested as an increased willingness to engage in deep processing of cognitive resources.

Accordingly, to ensure the effective implementation of EKT, depends on the joint effects of internalization and SM. In this process, explicit knowledge is reconstructed into a critical foundation for achieving cognitive transitions.

#### 2.3.4 Tacit-explicit knowledge integration: trust in AI (TiAI)

According to Chin et al., 2025, the conversion between tacit and explicit knowledge is not aimed at opposition per se, but at achieving a dynamic balance through their ongoing interplay within the process of SECI. This logic can be mapped onto the context of HAC, where the essence of AI-HI integration lies in a state constructed through opposition, contradiction, and tension, while continuously negotiating dominance and complementarity between humans and human-like agents. As argued by Siaw and Ali (2024), humans are better positioned to substitute for AI in external environmental scanning to identify opportunities, whereas AI primarily contributes through internal scanning based on data analysis. This perspective reveals a clear division of labor between human and human-like agents, with their complementarity grounded in a human-centered foundation.

In this sense, human TiAI constitutes a critical mediator that facilitates AI-HI integration. AI functions primarily as an amplifier for making tacit knowledge explicit, while human intelligence assume responsibility for judgment and selection in the process of rendering explicit knowledge tacit. Only when individuals maintain a calibrated relationship with AI, positioned between reliance and control, can human intelligence and AI achieve a systematic integration.

### 2.4 Research questions

Drawing on knowledge conversion to examine HAC in higher education, this study adopts a processual, configurational, and harmonizing perspective of TKE and EKT, and accordingly proposes the following research questions:

*RQ1*: In the context of HAC in higher education, through what combinations of SI, TKA, Internalization, SM, and TiAI (ET/ FT) can cognitive transition (or HLC) be achieved?

*RQ2*: How do these key psychological processes, across different cognitive transition pathways, form diverse configurational patterns through substitutive and complementary mechanisms?

*RQ3*: How do TiAI and its calibration function as regulatory mechanisms in the process of knowledge conversion, thereby shaping cognitive transitions under human-AI symbiosis?

*RQ4*: In HAC, is cognitive transition driven by a dominant psychological process, or does it emerge from the configuration of multiple interacting processes?

## 3 Methods

### 3.1 Research context and sample analysis

This study was authorized under a university-level project (Project No. A2023003), and all data collection and analysis followed the guidelines of the university ethics committee and teaching research standards. Within the scope of project authorization, the research context was selected from management-related courses, including *Market Research and Forecasting*, *Smart Tourism Practices*, and *SPSS Statistical Experiments*. The core tasks of these courses focus on digitally driven market data management and operational simulation, emphasizing students’ appropriate use of modern technologies for problem solving within project-based learning.

Across these courses, a shared assessment task—*City Walk Design*—was adopted. Students were required to use social network analysis tools (e.g., Ucinet, Pajek), geographic information systems (e.g., MapInfo), statistical software (e.g., SPSS, ChatSPSS), and large language models (e.g., ChatGPT, DeepSeek) to analyze spatial associations, route planning, and tourism experiences. This assessment task is characterized by a high degree of contextual complexity and openness. As such, it provides an appropriate and representative analytical setting for examining the mechanisms of HLC in HAC.

In terms of sample selection, this study adopts a cluster sampling approach, encompassing students who participated in the relevant course tasks. All included participants were required to complete a designated core task. The sample size of this study was 316, drawn from two cohorts of undergraduate students (2024–2025) majoring in management-related disciplines. Data were collected in 2025, a period marked by the rapid development of AI technologies, particularly large language models. The widespread adoption of AI in educational settings during this time renders 2025 a critical juncture, providing an important real-world context for examining AI-HI integration and its implications for cognitive transitions.

### 3.2 Measurement description

#### 3.2.1 Condition variables

Drawing on the unity-of-opposites process embedded in the SECI model (Chin et al., 2025), this study conceptualizes HLC in HAC as three interrelated mechanisms: TKE, EKT, and tacit-explicit knowledge integration within the process of knowledge conversion. Based on the conversion mechanisms and underlying logic within this framework, and drawing on prior studies such as Zhang et al. (2025a) and Jiang et al., 2025a, relevant variables were selected to correspond to the key psychological constructs involved in these processes.

The logical definitions of variable construction, conversion mechanisms, number of items, variable names, and reference sources are presented in Table 1, while the detailed measurement items are provided in Supplementary Table S1. In addition, it should be noted that, in order to better align with the specific context of this study, the variables SI, TKA, I, and SM were moderately contextualized.

**Table 1:** Construction of conditional variables.

SECI mechanism	Psychological dimension	Construct	items	Reference
Tacit-to-explicit conversion (S & E)	Context and trigger	Social interaction (SI)	6	Zhang et al., (2025a)
	Prior cognitive state	Tacit knowledge acquisition (TKA)	5	
Explicit-to-tacit conversion (C & I)	Core conversion	Internalization (I)	5	
	Psychological adjustment	Self-motivation (SM)	8	
AI-mediated SECI dynamics	Trust in AI	Functional trust (FT)	3	Jiang et al. (2025a)
		Emotional trust (ET)	3	

#### 3.2.2 Outcome variable

Drawing on Palmer and Devitt (2007), this study introduces a composite assessment approach that integrates performance-based tasks with course project outcomes to quantify the acquisition of HLC. Based on Bloom’s taxonomy, we link the dimensions of analysis, evaluation, and creation in course project performance to manifestations of higher-order cognitive outcomes. Specifically, key elements of the *City Walk design* task, such as multi-tool integration and the rationality of decision-making, are mapped onto the three forms of HLC. Within this context, graded evaluations are conducted according to criteria including depth of application, decision logic, contextual fit, and solution originality. Detailed descriptions of these evaluation criteria and their classification standards are provided in Supplementary Table S2.

It is important to note that the design and operationalization of the outcome variable in this study build upon our prior work (Jiang et al., 2025a, 2025b). In earlier studies, cognitive levels were measured and validated using the After Action Review (AAR) approach. The results demonstrated strong reliability and convergent validity of the measurement scheme, with indicators such as KMO = 0.83, total variance explained = 77%, AVE = 0.77, and CR = 0.93 (Jiang et al., 2025b). Building on this foundation, the present study extends this measurement framework to the higher education context, providing a continued validation and refinement of cognitive mechanisms in HAC.

#### 3.2.3 Convergence and explanatory power of variables

All condition variables were measured using questionnaire-based scales; therefore, their convergence and explanatory power were examined (Pappas and Woodside, 2021). The relevant results are reported in Table 2. The KMO values for all constructs reach or exceed 0.60, indicating an acceptable level and suitability for factor analysis. In addition, the total variance explained by each construct ranges from 42 to 57%, meeting the commonly accepted minimum standards in empirical research and suggesting that the indicators exhibit adequate explanatory potential for their respective constructs.

**Table 2:** Validity, convergence and interpretation power among conditional variable indicators.

Variable	items	KMO	Var. explained	AVE	CR
SI	6	0.82	45%	0.5	0.84
TKA	5	0.72	42%	0.4	0.8
I	5	0.78	50%	0.5	0.81
SM	8	0.84	54%	0.4	0.8
FT	3	0.6	57%	0.6	0.8
ET	3	0.6	57%	0.5	0.8

In terms of convergence, the AVE values of most variables approach or reach the threshold of 0.50, with a small number falling slightly below this criterion. However, when considered in conjunction with the CR results, the indicators demonstrate an overall acceptable level of convergent validity.

### 3.3 The fsQCA method

#### 3.3.1 The logic of fsQCA appropriateness

This study employs fuzzy-set qualitative comparative analysis (fsQCA) to examine the mechanisms underlying the formation of HLC in HAC contexts. The analytical logic of fsQCA emphasizes multiple pathways to outcomes and configurations of conditions, enabling the simultaneous examination of synergistic, substitutive, and asymmetric relationships among multiple key conditions. Unlike LRs or SEMs, fsQCA does not rely on explaining the net effect of a single variable; rather, it emphasizes how different configurations of condition variables can lead to the same outcome. This logic of model construction aligns closely with the theoretical framework of this study, which conceptualizes dynamic balance and imbalance mechanisms in HAC through the configuration of multiple psychological processes that are both oppositional and unified.

From the perspective of model construction requirements, the sample size in this study is moderate and meets the requirements for fsQCA (Vis, 2012). At the same time, the number of condition variables derived from the theoretical framework is appropriately specified, and these variables exhibit complex interactive relationships. With a single outcome variable as the analytical focus, this research design not only meets the requirements for comparative analysis of complex causal conditions but also effectively avoids the problem of configurational path explosion (Pappas and Woodside, 2021).

#### 3.3.2 Calibration principles and K-means

Calibration of the dataset is a critical step in fsQCA, and differences in data types can substantially affect the specification of calibration thresholds. The variables in this study mainly fall into two categories: questionnaire responses and user performance (the calibration principles and results are reported in Table 3). The condition variables SI, TKA, I, SM, FT, and ET are all questionnaire-response-based measures.

**Table 3:** Calibration of outcome variables and conditional variables.

Variables	Calibration			Thresholding	Reference
	Full	CP	Non		
SI	4.75 (95%)	2.5 (50%)	0.25 (5%)	Prior knowledge	Feng et al., (2026)
TKA					
I					
SM					
FT					
ET					
HLC	81.82	73.7	63.32	K-means	Jiang et al., (2025c)

Following established practices in prior research (Pappas and Woodside, 2021; Feng et al., 2026), the calibration anchors for mapping the data into the [0,1] set membership space were set at 95, 50, and 5%. These correspond to scale values of 4.75 (full membership), 2.5 (crossover point), and 0.25 (full non-membership), respectively, based on the five-point Likert scale ranging from 1 to 5 (strongly disagree to strongly agree).

The outcome variable (higher-level cognition, HLC) is a user performance-based measure. Its dataset calibration follows established practices in prior research (Jiang et al., 2025c), namely determining thresholds for user performance data through the machine-learning K-means clustering method. As an unsupervised learning approach (Sinaga and Yang, 2020), K-means is primarily used for grouping and pattern partitioning (Likas et al., 2003). Based on this approach, the fuzzy-set classification results are specified using three thresholds: full non-membership, the crossover point, and full membership. $X=\{x_{1},x_{2}\cdots ,x_{n}\}$ is the user performance dataset, the minimum total sum of squared errors is calculated as follows 1:


$$\min_{c}\sum_{k=1}^{3}\sum_{x_{i}\in C_{k}}{\left\|x_{i}-\mu_{k}\right\|}^{2}$$ (1)

Where $C_{k}$ denotes the k -th cluster, and $\mu_{k}$ represents the centroid (mean) of the k -th cluster. The centroids of the three clusters are then ordered as follows: $x_{0}=\mu_{\mathrm{low}}$ , $x_{0.5}=\mu_{\mathrm{mid}}$ and $x_{1}=\mu_{\text{high}}$ .

Finally, a standard logistic membership function is applied to transform the original variable x into fuzzy-set membership scores 2 (Sinaga and Yang, 2020), where α controls the slope of the curve.


$$f(x)=\frac{1}{1+\exp(-\alpha (x-x_{0.5}))}$$ (2)

## 4 Fuzzy-set QCA findings

### 4.1 Results of the necessity analysis

Table 4 reports the results of the necessity analysis for both the acquisition of higher-level cognition (HLC) and its non-acquisition (~HLC). The results show that SI, TKA, I, SM, ET, and FT all exhibit consistency levels exceeding 0.90 under conditions where HLC is achieved, with coverage values generally above 0.62. The results indicate that these conditions meet the threshold for necessity. However, caution is warranted in interpretation, as these same conditions also exhibit relatively high consistency when ~HLC is treated as the outcome, suggesting their strong prevalence within the sample. In this sense, “necessity” in the present study is better understood as a form of participatory condition rather than a causally decisive factor with high discriminative power. Accordingly, these conditions should be interpreted as necessary but not sufficient foundational elements in the formation of HLC.

**Table 4:** Analysis of necessity.

Conditions	HLC		~HLC	
	Consistency	Coverage	Consistency	Coverage
SI	0.95	0.62	0.94	0.48
~ SI	0.21	0.81	0.26	0.79
TKA	0.94	0.62	0.94	0.48
~ TKA	0.21	0.83	0.25	0.76
I	0.95	0.62	0.94	0.48
~ I	0.20	0.82	0.25	0.79
SM	0.95	0.62	0.94	0.48
~ SM	0.19	0.81	0.24	0.80
FT	0.96	0.63	0.92	0.47
~FT	0.20	0.76	0.28	0.83
ET	0.96	0.62	0.93	0.47
~ET	0.19	0.77	0.26	0.83

### 4.2 Configuration analysis results

Table 5 presents five configurational pathways identified through the fsQCA analysis, yielding an overall solution consistency of 0.773 and a solution coverage of 0.325. This indicates that these configurations are able to stably explain approximately 30% of the cases, which is consistent with expectations of equifinality and the coexistence of multiple pathways in complex contexts.

**Table 5:** Configurations for high-level cognitive acquisition in human-AI collaboration.

Conditions	C1	C2	C3	C4	C5
SI	●	●	●	●	●
TKA	●	●	●	●	X
I	●	●	●	X	●
SM	●	●	X	●	●
FT		X	●	●	●
ET	X		●	●	●
Raw coverage	0.188	0.200	0.194	0.198	0.214
Unique coverage	0.017	0.021	0.010	0.014	0.026
Consistency	0.775	0.765	0.817	0.826	0.834
Overall coverage	0.325				
Overall consistency	0.773				

Frequency cutoff = 1; Consistency cutoff = 0.77.

● = presence of contributing (peripheral) conditions.

X = absence of core conditions.

Blank spaces indicate “do not care” (the condition can be either present or absent).

From a holistic perspective, no core condition was identified across the configurations, further suggesting that the examined conditions operate primarily as necessary rather than sufficient conditions for the formation of HLC.

From a frequency perspective, SI appears in all identified configurations; however, it is not sufficient to independently constitute a decisive condition for the emergence of HLC. In contrast, TKA, internalization (I), and SM alternate between presence and absence across different configurational pathways, indicating that HLC does not rely on a single psychological mechanism. This finding supports the interpretation that substitutive and complementary relationships exist among these mechanisms.

Second, FT and ET do not co-occur across all configurations. This suggests that TiAI does not exhibit universal necessity; rather, its role is better characterized as a configurationally contingent regulatory mechanism. Such a role should be interpreted within the framework of configurational analysis.

To further assess robustness, additional analyses were conducted by setting the frequency cutoff to 1 and the consistency cutoff to 0.78 (Feng et al., 2026). The resulting configurational solutions are highly consistent with those reported in Table 5 (see Supplementary Table S3 for details), indicating that the findings are robust and stable.

### 4.3 Configurational induction

#### 4.3.1 Cognitive tension pathways under low-trust configurations

In C1 and C2, TiAI does not consistently emerge, despite the presence of other conditions at relatively high levels. From the perspective of knowledge conversion in HAC, these pathways can be interpreted as reflecting strong cognitive tension between tacit and explicit knowledge, in the absence of effective external regulatory mechanisms. Specifically, FT and ET are alternately absent across C1 and C2, while the remaining condition variables continue to exhibit high levels of engagement. This indicates that HLC can still be triggered under conditions of limited reliance on AI, primarily through intensive group interaction and contextual enhancement.

It should be further noted that the above interpretation is derived from theoretical extension. The configurational results of fsQCA merely indicate that alternative pathways to HLC exist even under low levels of TiAI, but do not directly uncover the underlying cognitive mechanisms.

Combined with teaching practice, this mechanism is clearly observed in the *City Walk design* context. When students identify discrepancies between routes generated by ChatGPT or DeepSeek and real urban conditions, cognitive tension arises within the group. As TiAI is reduced, team members rely more heavily on sustained discussion and experiential correction, thereby activating a forced reorganization of cognitive structures.

A comparison between the fsQCA results and teaching practice reveals that AI is not a necessary precondition for the emergence of HLC. In contexts where AI-driven support is absent or reduced, individuals can still promote the updating and restructuring of cognitive structures through iterative comparison and adaptive adjustment.

#### 4.3.2 Compensatory pathways of AI

Compared with C1 and C2, C3, C4, and C5 exhibit a stable presence of TiAI. Under conditions where both functional and emotional support from AI are available, the configurations of human-centered conditions are not identical. These findings suggest that TiAI can play a compensatory role under specific condition gaps.

From the perspective of knowledge conversion, AI provides informational feedback and support that help regulate constraints arising in the processes of TKE and EKT. When TKA is absent, C5 can be interpreted as obstacles in the TKE; when I and SM are absent, C3 and C4 indicate obstacles in the EKT.

Under these conditions of imbalance, the calibration of TiAI can be understood as an externally embedded regulatory mechanism that enables the re-coordination of tacit and explicit knowledge, thereby sustaining the continuity of the knowledge conversion process.

Combined with teaching practice, this mechanism is concretely manifested in the following ways.

*AI as a compensatory mechanism for impaired TKE*. Students possess abundant spatial vector data and statistical outputs and demonstrate basic operational proficiency. However, when entering the solution design stage, they often fail to grasp the underlying coupling logic embedded in the data, preventing meaningful enhancement of *City Walk design* solutions and user experience. When these data are imported into ChatGPT or DeepSeek, AI-assisted interpretation compensates for the absence of TKA, thereby facilitating the successful externalization of tacit knowledge.

*AI as a compensatory mechanism for impaired EKT*. During the *City Walk design* process, some students appear to interact frequently with AI, producing a large number of seemingly professional drafts. Yet these behaviors are primarily oriented toward task completion rather than problem solving, indicating the absence of SM. Moreover, once detached from AI support, the coupling mechanisms underlying the data remain cognitively inactive, reflecting a lack of internalization. In such cases, AI-generated structured checklists, such as task schedules and rigid evaluation criteria—provide a form of forced path dependence, ensuring that instructional tasks can continue to operate even under conditions of cognitive imbalance, and supporting the internalization of explicit knowledge.

By comparing the fsQCA results with teaching practice, it becomes evident that AI does not determine the direction of cognitive development. Instead, through complementary configurations with human-centered conditions, AI functions to regulate and extend human behavior, vision, and metacognitive boundaries.

#### 4.3.3 Integrative explanation

Integrating the two types of configurational pathways, the findings indicate that the emergence of HLC in HAC does not rely on a single, stable optimal pattern. Instead, it is characterized by multiple pathways arising from different combinations of conditions. Some configurations emphasize the dominance of human-centric factors, while others highlight the compensatory role of technological support in educational contexts. Together, these insights constitute an overarching explanatory framework for understanding the mechanisms of HLC in HAC.

## 5 Discussion

This study adopts a knowledge conversion perspective to examine the mechanisms of hlc in HAC. By integrating the configurational pathways identified through fsQCA with analytical interpretation, the findings provide substantial empirical responses to the proposed research questions.

First, in response to RQ1, the results indicate that HLC does not arise from any single psychological variable but is instead supported by the joint influence of multiple conditions. Methodologically, the principle of equifinality inherent in fsQCA offers an appropriate lens for understanding this phenomenon (Pappas and Woodside, 2021). Compared with traditional linear approaches (Georganta and Ulfert, 2024), this study demonstrates that knowledge conversion in HAC is better characterized as a multi-factor, coupled process. Otherwise, compared with recent extensions of the SECI model, Zhang et al. (2025a) emphasize the joint effects of TKA, SI, I, and SM on academic advancement. Building on this line of research, the present study further argues that knowledge conversion should not only be understood from a holistic perspective but also examined through the lens of dynamic oppositional relationships among key psychological elements.

Building on the response to RQ1, this study further argues that different psychological processes exhibit both substitutability and complementarity in HLC, thereby directly addressing RQ2. Unlike prior studies that primarily emphasize the positive effects of specific factors (e.g., Zhang et al., 2025a), their moderating or mediating roles (Li et al., 2025; Yang et al., 2025), or assume that these conditions must simultaneously coexist (Jiang et al., 2025b), the present study adopts a configurational perspective.

The fsQCA results indicate that, under no-AI configurations, high levels of investment in SI, TKA, I, and SM can be jointly activated, allowing human-centered cognitive tension to become the primary driving force of HLC. By contrast, under AI-compensated pathways, AI provides structural support for HLC by compensating for deficiencies in human-centered conditions through functional and emotional assistance.

More importantly, the empirical findings of this study support the dynamic process of contradiction, conflict, and reconciliatory conversion in HAC (Chin et al., 2025), thereby extending the explanatory boundaries of psychological perspectives in this domain.

Compared with prior studies (Jiang et al., 2025a), this research offers a more contextualized interpretation of TiAI. Our findings suggest that TiAI is not universally a necessary condition for achieving HLC; rather, its more critical role lies in mediation and compensation. This conclusion directly responds to RQ3, indicating that TiAI can compensate for the absence of specific human-centered conditions, thereby enabling HLC.

Within the context of higher education, when conversion imbalance emerges, the calibration of TiAI can function as an important form of external cognitive embedding. Conversely, when human-AI interaction has smoothly entered the process of knowledge spiral, a relative absence of TiAI may instead strengthen the social dynamics of the group.

These findings meaningfully revise the prevailing metaphorical assumption that “better calibration of TiAI inevitably leads to higher task effectiveness” (Huang and Rust, 2024), and further contribute to ongoing debates concerning cognitive inhibition resulting from overreliance on AI (Buçinca et al., 2021).

The results of the necessity analysis (Table 4) and the configurational analysis (Table 5) provide the most direct response to RQ4: there is no single, stable necessary condition in the process of achieving HLC. This conclusion is consistent with prior findings in HAC research, which suggest that no “universal prerequisite” exists (Chowdhury et al., 2022).

However, from the perspective of knowledge conversion, this process can be understood as comprising three interwoven dimensions:

first, the coexistence of embeddedness and substitutability of AI in cognitive processes;

second, the juxtaposition of ontological deficiency and high levels of human engagement;

and third, the inherent psychological tension embedded in the conversion between explicit and tacit knowledge.

### 5.1 Theoretical contributions

The primary theoretical contribution of this study lies in integrating the concept of knowledge conversion with contemporary approaches to psychological process analysis, thereby constructing an explanatory and empirically operationalizable framework for HAC. It should be noted, however, that this framework builds upon the unity-of-opposites perspective embedded in the SECI model (Chin et al., 2025) rather than offering a fundamentally novel theoretical reconstruction.

Specifically, drawing on recent quantitative extensions of SECI (Jiang et al., 2025a; Zhang et al., 2025b), this study operationalizes the unity-of-opposites elements in HAC as a set of measurable psychological process variables. Building on this, fsQCA is employed to examine necessity conditions and configurational pathways within the knowledge conversion process, transforming a predominantly theoretical perspective into empirically testable mechanisms of cognitive emergence. Furthermore, within the context of higher education, the study categorizes human–AI knowledge conversion into distinct patterns (e.g., transformation, balance, and imbalance), thereby enhancing contextual explanatory power.

Methodologically, this study also offers meaningful contributions. As noted by Pappas and Woodside (2021), much of the existing fsQCA literature remains at the level of pathway identification, with limited engagement in interpreting configurational logic and dynamic interdependencies, and insufficient integration with theoretical frameworks. Addressing this gap, the present study emphasizes the alignment between configurational patterns and theoretical logic, thereby advancing the integration of method and theory. Nevertheless, it is important to acknowledge that fsQCA is inherently a static analytical tool; thus, interpretations of dynamic processes are grounded in theoretical reasoning and empirical inference rather than direct observation.

Finally, in terms of research context, this study situates its analytical framework within HAC in higher education, offering a more nuanced and dialectical understanding of AI’s role in cognitive processes. Existing studies on technology-supported human development often adopt a relatively narrow, tool-centric perspective on AI, particularly in educational settings (Herrmann-Werner et al., 2024). In contrast, this study highlights the multifaceted roles of AI in HAC, including supportive, compensatory, and substitutive functions, thereby enriching our understanding of AI’s role in cognitive development.

### 5.2 Educational implications

As discussed above, this study demonstrates that, within HAC contexts in higher education, the realization of cognitive transitions does not necessarily depend on the functional or emotional support provided by AI. Across different task contexts, such transitions are instead contingent upon configurations of multiple psychological processes that are inherently contradictory.

When social factors, metaphorical scaffolding, self-regulation, and absorptive capacity serve as critical antecedent conditions for cognitive transitions, such transitions may be achieved either through the generation of cognitive tension in the absence of TiAI, or through structural supplementation via the calibration of AI to sustain cognitive continuity. Accordingly, this study argues that AI should not be conceptualized merely as an efficiency-enhancing tool or a substitute for human cognition. Rather, its substantive value in higher education lies in repairing cognitive imbalance and providing stable support throughout the learning process.

This study further demonstrates that the effective operating mechanisms of HAC vary substantially across different instructional tasks and learning stages. Using the *City Walk design* assignment as an illustrative case, this study examines a task characterized by high complexity and openness, rendering it particularly representative. Within this task context, when AI-generated outputs conflict with learners’ existing cognitive structures, such discrepancies may instead function as critical triggers for group discussion and meaning reconstruction. Under these conditions, a reduction in TiAI does not impede learning progress; rather, it strengthens human agency and subjectivity.

By contrast, when misalignment arises between learners’ human-centered conditions and task complexity, AI provides functional and emotional support that helps regulate cognitive load and sustain learning engagement. Taken together, these findings indicate that the practical effectiveness of HAC is highly contingent upon task characteristics, learner conditions, and instructional design.

From an instructional implementation perspective, this study argues against both the indiscriminate adoption of AI and the intentional restriction of its use. Our findings suggest that the degree to which AI should be integrated depends critically on the instructional stage and the situational characteristics of HAC.

During the phase of cognitive tension generation, allowing AI to produce uncertain or even conflicting outputs can activate collaborative engagement between the self and others. In contrast, during the phase of cognitive reconciliation, calibration of AI becomes necessary to sustain learning continuity. The core instructional strategy, therefore, lies in avoiding the risk of cognitive outsourcing caused by excessive reliance on AI, while simultaneously preventing technological exclusion that may foreclose opportunities for cognitive transition.

## 6 Limitations

It must be acknowledged that this study is subject to certain limitations; however, these constraints provide clear avenues for further resolution in future research.

The practical implications of this study are primarily derived from management-oriented and application-driven course contexts. The generalizability of the findings across different disciplines, cultural backgrounds, and educational systems therefore requires further empirical validation.

Also, this study mainly focuses on student-level psychological mechanisms. Due to constraints related to article length and the limitations of fsQCA in modeling actor-based regulatory roles, the moderating and facilitative functions of teachers were not systematically examined. However, in educational contexts characterized by deep AI integration, the role of teachers is undergoing a fundamental transformation (Fan et al., 2025). Teachers are no longer the ultimate arbiters of learning outcomes, nor merely buffers correcting students’ misjudgments. Instead, their role in guiding, triggering, and regulating knowledge spiral in learning processes warrants more explicit theoretical articulation and empirical investigation in future research.

## Data Availability

The raw data supporting the conclusions of this article will be made available by the authors, without undue reservation.

## Footnotes

1 SECI refers to the knowledge spiral model.
