# Supplementary material for: Beyond facilitation and inhibition: a configurational mechanism study of cognitive transitions in human–AI collaboration
Source: Front Psychol. 2026 Apr 29;17:1821188. doi: 10.3389/fpsyg.2026.1821188 (PMC13167932; doi:10.3389/fpsyg.2026.1821188)
Supplement: Supplementary file 1 [file Supplementary_file_1.DOCX]

Supplementary Material

**Table S1. Scale Design for Conditional Variables**

| Transformation Mechanism | Dimensions | First-order Constructs | Items | Reference |
| --- | --- | --- | --- | --- |
| TKE | Context and Triggering Mechanism | Social Interaction (SI) | SI1: With the support of AI tools, we maintain close and continuous academic interactions with our professors. | Zhang et al., 2025 |
|  |  |  | SI2: With the help of AI tools (e.g., generation, analysis, or collaboration functions), our team members frequently interact with other teams. |  |
|  |  |  | SI3: During human-AI collaboration, our team members learn together and exchange new ideas with AI. |  |
|  |  |  | SI4: We interact with AI and hold regular meetings to acquire new knowledge and perspectives. |  |
|  |  |  | SI5: In AI-supported learning or research processes, we regularly communicate with teachers and share research progress. |  |
|  |  |  | SI6: When using AI tools for learning tasks, we still maintain close social relationships with team members. |  |
|  | Pre-existing Cognitive State | Tacit Knowledge Acquisition (TKA) | TKA1: In lectures and seminars assisted by AI tools, we can obtain a large amount of non-codified (tacit) academic knowledge. |  |
|  |  |  | TKA2: Through AI-supported communication methods, we can acquire non-codified academic knowledge by interacting with teachers. |  |
|  |  |  | TKA3: In the process of collaborating with classmates and AI, we can obtain non-codified academic knowledge. |  |
|  |  |  | TKA4: Through group meetings or collaborative activities combined with AI tools, we are able to acquire non-codified academic knowledge. |  |
|  |  |  | TKA5: In an AI-supported learning environment, self-motivation enables me to acquire non-codified academic knowledge. |  |
| EKT | Core Conversion Process | Internalization (I) | I1: In AI-supported learning environments, members regularly receive and practice training or guidance related to teamwork principles. |  |
|  |  |  | I2: In regular meetings combined with AI tools, we discuss the progress of high-quality academic research. |  |
|  |  |  | I3: Strategic plans for learning and projects were developed with the support of AI-assisted data analysis and feedback. |  |
|  |  |  | I4: Acquiring knowledge through internalization activities involving AI can improve our academic achievement. |  |
|  |  |  | I5: Engaging in internal intellectual pursuits with AI participation helps us learn more knowledge externally. |  |
|  | Psychological Moderation | Self-Motivation (SM) | SM1: I will be able to achieve most of the goals I set for myself. |  |
|  |  |  | SM2: When facing complex tasks, I am confident that I can complete them. |  |
|  |  |  | SM3: Overall, I believe I can achieve outcomes that are important to me. |  |
|  |  |  | SM4: I believe I can succeed in almost anything I set my mind to. |  |
|  |  |  | SM5: I will be able to overcome many challenges. |  |
|  |  |  | SM6: I am confident in performing well on many different tasks. |  |
|  |  |  | SM7: Compared to others, I can complete most tasks very well. |  |
|  |  |  | SM8: Even when situations are difficult, I can perform quite well. |  |
| Tacit-Explicit Knowledge Integration | Trust in AI | Functional Trust (FT) | FT1: The intelligent technology operates well and can effectively complete the tasks I require. | Jiang et al., 2025 |
|  |  |  | FT2: The intelligent technology possesses the functions necessary to complete key tasks. |  |
|  |  |  | FT3: The intelligent technology is competent within its field of expertise. |  |
|  |  | Emotional Trust (ET) | ET1: I think AI behaves like a human when interacting with me. |  |
|  |  |  | ET2: I think the mental effort required to interact with AI is reasonable. |  |
|  |  |  | ET3: I have an emotional dependence on the AI social service robot. |  |

**Table S2. Quantification of Outcome Variables**

| Dimensions | Type of Higher-order Cognition | 60–70 (Low) | 71–81 (Medium) | 82–100 (High) |
| --- | --- | --- | --- | --- |
| Spatial-Behavioral Analytical Capability | Analysis | Route design is primarily based on intuition or simple descriptions. | Able to explain by incorporating partial GIS or network data. | Capable of systematically analyzing spatial distribution, nodal relationships, and tourist behavior logic. |
| Multi-tool Integration Capability (AI + GIS + SNA + Statistical Tools) | Analysis | Tools are used in isolation, lacking logical connection. | Able to initially integrate the results from different tools. | Able to clearly explain the complementary roles of various tools in the cognitive process. |
| Rationality of Route Decision-making | Evaluation | Lacks a clear basis for design. | Able to explain some of the reasons for decisions. | Capable of comparing and demonstrating design schemes based on data and context. |
| Contextual Transfer and Application Capability | Application | Design is detached from real urban contexts. | Partially matches real-world contexts. | Fully considers tourist experience, urban characteristics, and implementation feasibility. |
| Innovation and Scheme Uniqueness | Creation | Significant homogeneity in route designs. | Exhibits a degree of differentiated design. | Proposes new schemes that are distinctly different from conventional tourism routes. |

**Table S3. Robustness Check: Configurations for Higher-level Cognitive Acquisition**

| Conditions | C1 | C2 | C3 | C4 |
| --- | --- | --- | --- | --- |
| Social Interaction (SI) | ● | ● | ● | ● |
| Tacit Knowledge Acquisition (TKA) | ● | ● | ● | 🞮 |
| Internalization (I) | ● | ● | 🞮 | ● |
| Self-Motivation (SM) | ● | 🞮 | ● | ● |
| Functional Trust (FT) |  | ● | ● | ● |
| Emotional Trust (ET) | 🞮 | ● | ● | ● |
| Raw Coverage | 0.188 | 0.194 | 0.198 | 0.214 |
| Unique Coverage | 0.026 | 0.014 | 0.016 | 0.033 |
| Consistency | 0.775 | 0.817 | 0.826 | 0.834 |
| Overall Coverage | 0.304 |  |  |  |
| Overall Consistency | 0.787 |  |  |  |

Notes: Frequency cutoff = 1; Consistency cutoff = 0.78

● = presence of contributing (peripheral) conditions.

🞮 = absence of core conditions.

Blank spaces indicate "do not care" (the condition can be either present or absent).
